# Development of an Oral *Salmonella*-Based Vaccine Platform against SARS-CoV-2

**DOI:** 10.3390/vaccines10010067

**Published:** 2022-01-01

**Authors:** Wonsuck Yoon, Yongsung Park, Seunghyun Kim, Iel Soo Bang

**Affiliations:** 1 Allergy Immunology Center, Korea University, Seoul 02841, Korea; seunghyun@korea.ac.kr; 2 Air-Microbiome Health Research Institute, College of Medicine, Korea University, Seoul 02841, Korea; 3 Department of Life Science and Biotechnology, Korea University, Seoul 02841, Korea; dcomtrue@korea.ac.kr; 4 Department of Microbiology and Immunology, Chosun University School of Dentistry, Gwangju 61452, Korea; isbang@chosun.ac.kr

**Keywords:** oral vaccine, *Salmonella*, SARS-CoV-2, spike protein

## Abstract

Effective vaccine development for global outbreaks, such as the coronavirus disease 2019 (COVID-19), has been successful in the short run. However, the currently available vaccines have been associated with a higher frequency of adverse effects compared with other general vaccines. In this study, the possibility of an oral bacteria-based vaccine that can be safely used as a platform for large-scale, long-term immunization was evaluated. A well-known *Salmonella* strain that was previously considered as a vaccine delivery candidate was used. Recombinant *Salmonella* cells expressing engineered viral proteins related with COVID-19 pathogenesis were engineered, and the formulation of the oral vaccine candidate strain was evaluated by in vitro and in vivo experiments. First, engineered S proteins were synthesized and cloned into expression vectors, which were than transformed into *Salmonella* cells. In addition, when orally administrated to mice, the vaccine promoted antigen-specific antibody production and cellular immunity was induced with no significant toxicity effects. These results suggest that *Salmonella* strains may represent a valuable platform for the development of an oral vaccine for COVID-19 as an alternative to tackle the outbreak of various mutated coronavirus strains and new infectious diseases in the future.

## 1. Introduction

The global outbreak of the coronavirus disease 2019 (COVID-19) has increased the need for rapid vaccine development [1,2,3,4]. Since the COVID-19 outbreak, which is caused by the novel severe acute respiratory syndrome coronavirus 2 (SARS-CoV-2), several vaccines have been developed through various platforms, including live-attenuated and inactivated vaccines (first generation), protein subunit and vector-based vaccines (second generation), and nucleic acid and nano-material-based vaccines (third generation) [5,6]. Despite the significant efforts made, researchers are still trying to find effective vaccine candidates to block the expansion of the coronavirus, thus suggesting that the currently available vaccines are not the best. To date, three types of vaccines based on the Spike (S) protein have been commercialized: mRNA-1273 from Moderna, AZD1222 from AstraZeneca, and BTN162 from Pfizer and BioNTech [7,8,9]. However, vaccine-related undesired side effects have been reported and specific antibody prevalence in the body is too short [8].

Information on the features of this coronavirus is still lacking, making vaccine production with traditional vaccine platforms (such as using live-attenuated virus) a challenge. Among the available development strategies, mRNA vaccines based on the genetic information of the virus strain rather than virus itself is now preferred and generalized as a new method for developing an anti-coronavirus vaccine [5,6,9]. However, in the case of the currently commercialized viral vector- and RNA-based vaccines, due to the short development period and limitations of clinical evaluation, some side effects have been reported. In particular, there are public concerns about the incidence of anaphylaxis, thrombosis with thrombocytopenia syndrome (TTS), Guillain–Barré syndrome, myocarditis, and pericarditis associated with these RNA- and virus-based vaccines [10], which are mainly due to the excessive activity of the immune system in response to the injection of excessive amounts of inflammatory substances directly into the body. Despite these negative aspects of the currently available COVID-19 vaccines, they are indispensable for managing and controlling the ongoing global pandemic. Under this circumstance, research on the development of new, rapid, and safer vaccine technology is necessary. Thus, bacterial-based orally administered vaccines have attracted attention as a safer vaccine technology compared with vascular or intramuscular injection approaches, and are also a rapid vaccine development platform for new infectious diseases such as COVID-19.

Human coronaviruses are positive-single RNA viruses that consist of the four types of structural proteins—S, envelope (E), membrane glycoprotein (M), and nucleocapsid (N) proteins [5]—of which the S protein has been used as a major antigen in the current commercialized vaccines owing its highly conserved receptor binding domain against the angiotensin-converting enzyme 2 (ACE2). Additionally, it is reported that the S protein induces the activation of immune responses. Notably, recent reports described E and ORF7a proteins as being related to the pathogenesis of COVID-19 [11], as the E protein can bind to the tight junction-associated PALS1 and ORF7a can interact with CD14^+^ monocytes [12]. These findings suggest vaccines targeting E and ORF7a proteins are warranted, as well as against the S protein.

Currently, RNA- and virus-based vaccines use lipid nanoparticles or adenoviruses as tools for increasing immunogenicity [6,13,14]. However, these adjuvants have some setbacks: lipid nanoparticles can be painful and cause discomfort, and have low bioavailability, whereas adenoviruses can cause headaches, fever, nausea, muscle pain, and joint pain. Thus, researchers are searching for alternative adjuvants with fewer side effects, such as the bacille Calmette–Guerin and vaccinia virus, among others [3,5,6]. *Salmonella* itself has not been used as a bacterial-based vaccine platform for producing neutralizing antibodies; however, it would be worthwhile to try since it can promote cellular immune responses [15,16].

This study aimed to investigate the possibility of developing an orally administered COVID-19 vaccine using synthetic antigens (modified S protein) along with recombinant bacteria, such as the already informed *Salmonella* strains.

## 2. Materials and Methods

### 2.1. Bioinformatics STUDIES and Vector Construction

The sequence of SARS-CoV-2 virus Spike gene was elicited from valid databases (e.g., https://www.gisaid.org/, https://nextstrain.org/, https://www.ncbi.nlm.nih.gov/) (asscssed on 18 July 2020) and SARS-CoV-2 S-2P, comprising proline substitutions at residues K986 and V987, was chosen as the target antigen. GFP gene sequence was employed to produce a reporter mRNA model. The coding region of GFP and S protein were designed and synthetized under the control of T7 bacteriophage promoter and the entire sequence was inserted within the pUC57 vector. Following the confirmation of sequences, they were transformed into the Stbl4 strain of *E. coli*, known to be useful for plasmid reception as well as enormous amplification of vectors.

### 2.2. Construction of Salmonella Expressing COVID-19 Ag

At first, the Envelope gene (E, protein_id = UFE14292.1), ORF7a gene (7a, protein_id = UFE14252.1), and modified Spike protein gene of SARS coronavirus 2 (accession number, OL583529) were cloned into pET14b vector with NdeI and BamHI: each gene was obtained through the gene synthesis and the constructed vectors were designated as pET14b-mbe, pET14b-mb7a, and pET14b-e.spike. In order to express each gene at the *Salmonella* strain (BRD509), T7 promoter region of pET14a vector was replaced with Lac promoter of pUC19 using the Gibson assembly cloning method [17] with the following primers: for Lac promoter cloning, 5′-GCGTAGAGGATCGAGATCtaatacgcaaaccgcctct-3′ and 5′-ATGATGGCTGCTGCCCATagctgtttcctgtgtgaaat-3′; for the inverse PCR primer to amplify the vector harboring E and 7a genes, 5′-AGATCTCGATCCTCTACGCCGG-3′ and 5′-aTGGGCAGCAGCCATCATCATCA-3′. The resulting plasmids were called pKU-Ag.e.spike. For the amplification of pKU-Ag.e.spike, it was extracted from the transformed *S. typhimurium* SF586 strain (SF586). Therefore, the plasmids were used to transform *S. typhimurium* BRD 509 (BRD 509) cells which are a mutant aroA/aroD variant of *Salmonella* strain SL1344.

### 2.3. Western Blot Analysis

The whole cell of bacterial culture was run on an SDS-PAGE and electrophoretically transferred to nitrocellulose membranes. The membranes were pre-equilibrated with TBS-T solution containing 5% skim milk overnight and were incubated with mouse anti-His antibody (Cell Signaling Technology, Catalog #2366) for 2 h. The membranes were incubated with goat anti-mouse IgG HRP conjugate for 40 min at room temperature. Immune reactive protein bands were visualized using BM chemiluminescence blotting substrate.

### 2.4. Invasion Assay

Murine macrophage cell line (RAW 264.7 cells) was cultured in Dulbecco’s modified Eagle’s medium (DMEM) with 10% fetal bovine serum (FBS) (Welgene Inc., Gyoungsan-si, Korea) supplemented with antibiotics (100 units/mL penicillin, 100 mg/mL streptomycin) (Sigma-Aldrich, St. Louis, MO, USA). RAW 264.7 cells were allowed to grow in the wells overnight, creating a flat layer. *Salmonella* was separately grown overnight. The next day, the RAW 264.7 cells were inoculated with the *Salmonella* at MOI of 100:1 and were incubated together for 1 h. Centrifuging the plates for a few minutes may help bring cells and *Salmonella* in contact and initiate infection. After that, the cells were washed 3 times with PBS and then treated with 100 µg/mL gentamycin (Invitrogen) solution for 1 h to kill *S. typhimurium* remaining outside the cells. The plates were then washed well to remove the dead bacteria. After that, RAW 264.7 cells were lysed using 1% Triton X-100 for 5 min at 37 °C to detect *Salmonella*. They were incubated on LB plates by plating 10-fold serial dilutions. Colony forming units (CFUs) were counted on the next day.

### 2.5. In Vitro Cytotoxicity 

Following treatment with the engineered *S. typhimurium*, the viability of RAW 264.7 cells was assessed by the trypan-blue dye exclusion assay and the lactate dehydrogenase (LDH) assay. The exclusion of trypan-blue dye by viable cells was evaluated under a light microscope using a hemocytometer. A minimum of 200 cells was counted. LDH activity was determined by measuring the release of LDH from the cytosol into the culture medium after membrane rupture, using a CytoTox 96 non-radioactive cytotoxicity assay kit (Promega) in accordance with the manufacturer’s protocols. *Salmonella*-induced LDH release was calculated as a percentage of the LDH in culture medium compared with the total LDH in the lysed cells plus the culture medium.

### 2.6. Cytokine Assay

After oral administration of *S. typhimurium* and genetically engineered *S. typhimurium* in Balb/c mice, sera were collected by centrifugation at 2000× *g* at 4 °C for 5 min. Samples from the same groups were pooled and analyzed using an IFN-γ sandwich enzyme-linked immunosorbent assay (ELISA) kit (Bender Medsystems Inc., Burlingame, CA, USA) according to the manufacturer’s instructions.

### 2.7. Measurement of Bacterial Accumulation in Normal Mouse Tissues after Oral Inoculation 

The isolation and titration of bacteria from normal tissues such as lung, spleen, liver, kidney, and blood. Briefly, mice that were orally infected (*n* = 5) with the genetically engineered *Salmonella* (1 × 10^7^ CFU each) were humanely euthanized, and their tissues were removed aseptically followed by homogenization in PBS. Serial dilutions of homogenates were spread onto LB agar plates and were incubated at 37 °C for 24 h. The viable bacterial titers were then determined by counting the colonies (CFUs) and multiplying with the dilution factor.

### 2.8. Animal Studies 

Specific pathogen-free Balb/c female mice were purchased from the Shizuoka Laboratory Animal Center (Hamamatsu, Japan). Animals 6 to 8 weeks old were used throughout the experiments. 

Mice were given unrestricted access to food and water. Groups of three to five mice were immunized with recombinant *Salmonella* expressing COVID-19 Ag candidates. For the bacterial immunization, mice were orally inoculated with 100 μL of 1 × 10^7^ colony-forming units (CFUs)/mouse *S. typhimurium* suspension and were also boosted twice with the same preparation at 2-week intervals. The control mice received an oral injection of PBS only.

### 2.9. Investigation of Humoral Immune Response

Serum samples were collected from mice by eye bleeding before immunization and 1 week after each immunization and analyzed for the presence of Spike protein-specific antibodies with subtyping. Microtiter plates were coated with 100 mL of a Spike protein (2 μg/mL) in carbonate–bicarbonate buffer (pH 9.6) and incubated for 1 h at 37 °C. The plates were washed three times and blocked for 1 h at room temperature with 5% skim milk in PBS. After three washes, diluted serum samples were incubated for 1 h at 37 °C. The plates were incubated with HRP-conjugated goat anti-mouse IgG (1:10,000; Jackson Immuno Research Laboratories), rat anti-mouse IgG1 (1:500; Boehringer Mannheim), or rat anti-mouse IgG2a (1:500; Boehringer Mannheim), and specific reactions were detected by tetramethyl benzidine as a substrate. The OD was measured at 450 nm following the addition of stop solution. The titers were determined as the reciprocal of the highest serum dilution of the OD value greater than that obtained with the pre-immune sera.

### 2.10. Investigation of Cell-Mediated Immune Response

Spleen cells were harvested 1 week after the last immunization and cocultured for 4 days at 37 °C with an identical number of naive spleen cells previously treated with mitomycin C at 10^6^ cells/mL in 24-well plates in the presence of Spike protein (NKMAX, Germany, Catalog# ATGP3962, 2 μg/mL), Following the culture, cells were washed twice with complete RPMI-5 and then used as effector cells. Syngeneic naive spleen cells were prepared by adsorption of Spike protein (2 μg/mL) for 3 days at 37 °C, washed three times with complete RPMI-5, and resuspended at a concentration of 5 × 10^6^ cells/mL for use as target cells. The pulsed targets were added in 100 mL volumes to 96-well microtiter plates at 1 × 10^4^ cells/well, and effector cells were then added in a 100 mL volume at the indicated E:T ratios in triplicate. After 4 h of incubation, antigen-specific cytolysis was measured by using the CytoTox96^®^ Non-Radioactive Cytotoxicity Assay (Promega) according to the manufacturer’s manual. The assay quantitatively measured the lactate dehydrogenase (LDH) released on cell lysis. The percentage of specific lysis was calculated as ((experimental-effector spontaneous-target spontaneous)/(target maximum-target spontaneous)) × 100.

### 2.11. Statistical Analysis 

All the obtained information was evaluated using GraphPad Prism 9 software. *t*-test and one-way ANOVA (followed by Tukey’s post-test) algorithms were used as required to analyze the results statistically. A *p*-value lower than 0.05 was considered a significant difference.

## 3. Results

### 3.1. Design of Orally Administrated, Bacteria-Based Anti-Coronavirus Vaccines

**Figure 1:**
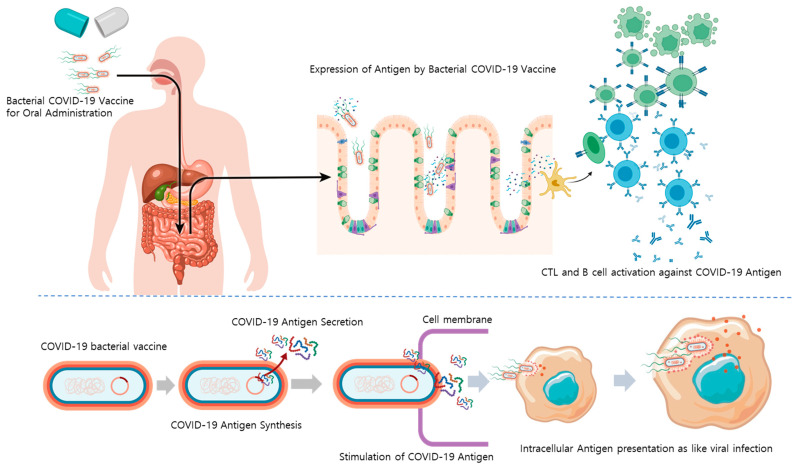
Mode of action of an orally administrated bacterial COVID-19 vaccine. Abbreviations: COVID-19, coronavirus disease 2019; CTL, cytotoxic T-cell.

When taken orally, bacteria strains that can tolerate the stomach and intestines can be used to develop bacteria-based vaccines. Herein, an attenuated *Salmonella* strain (with reduced virulence and toxicity) was explored as a vector for oral vaccines. This strain was designed to synthesize or partially secrete antigens against SARS-CoV-2 using the sipB160 protein. Importantly, this *Salmonella* can act as a vaccine by promoting B- and T-cells activation by phagocytosis or viral infection of macrophages resident in the intestine with good bacterial cell viability (Figure 1).

### 3.2. Construction of Salmonella Expressing COVID-19 Antigens

**Figure 2:**
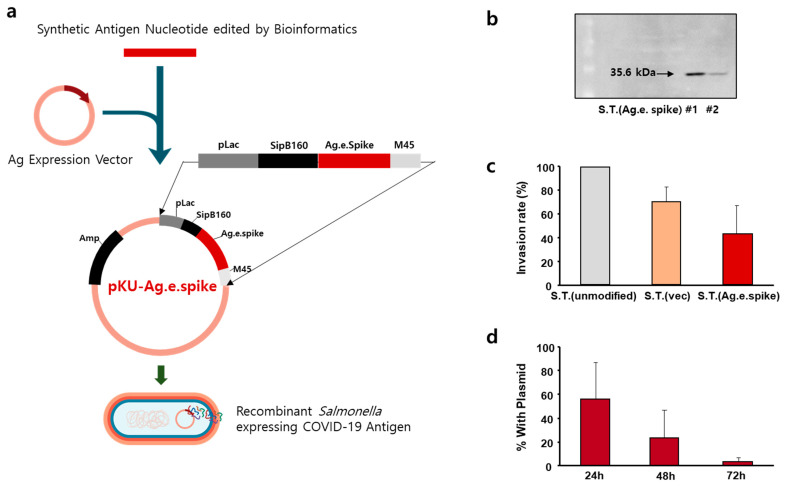
Construction of *Salmonella* Typhimurium expressing engineered spike protein as an antigen. (**a**) Schematic representation of the pKU-Ag.e.spike vector expressing engineered spike protein. (**b**) Expression of engineered spike protein in genetically engineered *S.* Typhimurium was measured by Western blot. (**c**) RAW 264.7 cells were infected with the indicated *S.* Typhimurium at a multiplicity of infection of 500:1 for 1 h (for invasion assay). The invasion rate of murine RAW 264.7 cells by the unmodified *S.* Typhimurium was set to 100%, and the relative internalization levels were normalized against those of unmodified *S.* Typhimurium. The data represent the mean ± standard error of three independent experiments. (**d**) Stability of pKU-Ag.e.spike in *S*. Typhimurium without antibiotic selection. Data represent the mean ± SD of a representative experiment.

Three types of antigens were synthesized based on bioinformatics data on the genetics of antigens that can be used for developing COVID-19 vaccines. As a vector capable of expressing an antigen in *Salmonella*, a plasmid using the lac operon was prepared. *SipB160*, which was edited with a signal peptide and bound to the antigen sequence, was used for granting the antigen secretion mechanism to the *Salmonella* model. The designed plasmid was synthesized and transformed into the bacteria. In this process, after confirming the cloning of the vector in *Escherichia coli*, the produced plasmid was transformed into a shuttle *Salmonella* strain and into a *Salmonella* strain that could be used as vaccine (Figure 2a). To confirm that the antigen was well expressed in the prepared vaccine candidate strain, three types of coronavirus protein antigen candidates were prepared. After culturing the strain, expression of engineered spike proteins was confirmed by Western blotting (Figure 2b) and expression of antigens of various sizes was also confirmed using this engineered strain (Supplement Figure S1). The characteristics of these bacteria strains were compared by measuring the ability of the bacteria to infect macrophages (Figure 2c). Compared with the unmodified strain, the infection rate was reduced by more than 50% in the strain transformed with the protein expression vector. In addition, evaluation of the expression of the vector during subculture confirmed that the bacteria lost all protein expression vectors within 72 h (Figure 2d).

### 3.3. Characteristics of Salmonella Expressing COVID-19 Antigen (Modified S Protein)

To measure the safety and stability of the recombinant coronavirus antigen-expressing *Salmonella* strain, in vitro and in vivo cytotoxicity after exposure for 24 h was evaluated.

**Figure 3:**
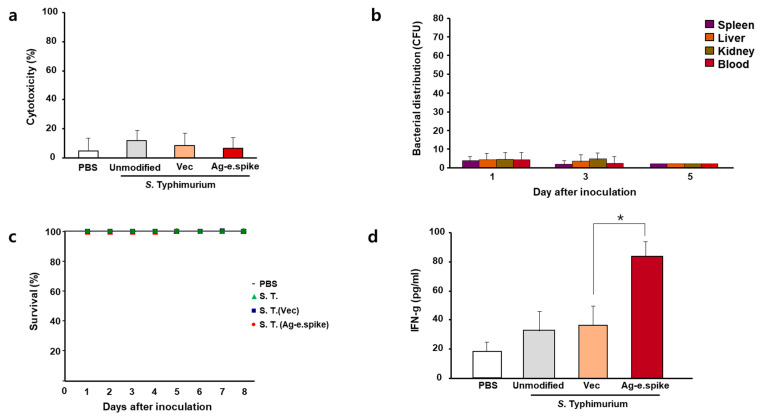
In vitro and in vivo effects of *S.* Typhimurium expressing Ag-e.spike. (**a**) LDH cytotoxicity assays were performed on cell-free supernatants from Raw 264.7 cells treated with phosphate-buffered saline (PBS) or attenuated unmodified *S.* Typhimurium, *S.* Typhimurium carrying the empty expression vector (vec), or *S.* Typhimurium expressing Ag-spike. The data represent the mean ± standard error of three independent experiments. (**b**) Bacterial distribution was examined in mice after oral administration 1 × 10^7^ bacterial cells for the indicated time. (**c**) Survival curves of all mice groups were recorded for up to 8 days. (**d**) Mice were treated with PBS or 1 × 10^8^ bacteria cells. Serum was collected 7 days after inoculation and interferon-γ (INF-g) levels were analyzed with enzyme-linked immunosorbent assay (ELISA). (* *p* < 0.05 versus *S.* Typhimurium (Vec)-treated mice; Student’s *t*-test).

Overall, only minor differences regarding toxicity were observed between in vitro cultured macrophages exposed to the different prototype engineered *Salmonella* strains (Figure 3a). Further in vivo experiments confirmed that the engineered bacteria did not accumulate in the body upon oral administration (Figure 3b) nor affected the survival rate of the mice. Nonetheless, overdose of orally live strains may affect survival (Figure 3c) and body weight change (Supplement Figure S2). Importantly, changes in levels of serum cytokines were not induced upon oral administration (Supplement Figure S3). In particular, interferon-γ was increased after the administration of the bacteria, with the antigen-expressing strain showing slightly higher levels of this cytokine (Figure 3d). 

### 3.4. In Vivo Induction of Anti-Viral Immune Responses after Oral Treatment with Salmonella Expressing COVID-19 Antigen (Modified Spike Protein)

**Figure 4:**
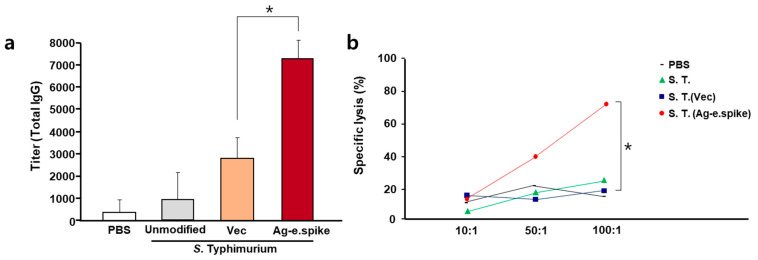
In vivo effects of oral treatment with *S*. Typhimurium expressing Ag-e.spike. Balb/c mice were orally inoculated on days 1, 14, and 28 with PBS or with 1 × 10^7^ CFU of attenuated unmodified *S.* Typhimurium, *S.* Typhimurium carrying the empty expression vector (vec), or *S.* Typhimurium expressing Ag-spike. (**a**) Mice were bled every 2 weeks, and total IgG antibody titer was determined by ELISA using equally pooled sera samples from each experimental group. (**b**) Comparison of S protein-specific CTL activities in BALB/c mice orally immunized with 10^7^ bacteria cells at 0, 2, and 4 weeks. One week after the last immunization, spleen cells were obtained and restimulated in vitro with the recombinant S protein-pulsed syngeneic stimulator cells. The specific cytolytic activity was tested against syngeneic targets in an LDH release assay. Spleen cells from unimmunized mice were used as control. Results represent mean specific lysis values from representative individual mice tested at the indicated effector:target cell ratios. (* *p* < 0.05 versus *S. typhimurium* (Vec)-treated mice; Student’s *t*-test).

Immune activity was evaluated in BALB/c mice after oral administration of 10^7^ bacteria three times at 2-week intervals. The production rate of antigen-specific antibodies in the serum was significantly increased (over 8000-fold) compared with the control group (Figure 4a). In addition, increased levels of IgG2a subtype antibodies, which are known to be produced by Type 1 immune responses, were also observed (Supplement Figure S4a). Therefore, to further assess the activity of cellular immunity, T-cell activation was measured. Antigen-specific activity of T-cells of immunized mice was confirmed (Supplement Figure S4b), and effective cellular immune response was detected in the experimental group treated with the orally administered *Salmonella* vaccine strains by antigen-specific cytotoxic T-cell (CTL) reaction (Figure 4b).

## 4. Discussion

In this study, engineered S proteins of SARS-CoV-2 were evaluated as potential therapeutic candidates for the prevention of COVID-19. These viral proteins were shown to trigger SARS-CoV-2-specific antibody production and activate COVID-19-specific cellular immune responses when administrated using *Salmonella* as transport platform. Moreover, orally administrated *Salmonella* BRD509 was shown to not have relevant cytotoxic effects in vivo. Hence, these in vitro and in vivo data suggest that *Salmonella* strains may represent a valuable new delivery system for COVID-19 vaccines.

The novel SARS-CoV-2 has quickly spread across 216 countries in 11 months, affecting more than 45 million people and being associated with a death rate of 1–3% worldwide [5,18,19]. Currently, there are no fully effective treatments for COVID-19; thus, fast vaccine development for this disease is still required.

To date, COVID-19 vaccines have been designed using various vaccine platforms, but only one mRNA-based vaccine (Pfizer and BioNTech) has been approved by the US Food and Drug Administration, while other vaccine types are still under phase I/II/III clinical trial [5]. Researchers have been continuously working to find an efficient vaccine for COVID-19. There are five main platforms to design the candidate vaccine: (1) live-attenuated, (2) mRNA vaccines, (3) DNA, (4) subunit, and (5) viral-vector-based vaccines [3,5]. Live-attenuated vaccines have been the most efficacious platform to ensure long-lasting neutralizing antibody production and immune responses; however, there are the several concern on the reviving virulence of the used delivery microorganism. DNA, mRNA, and subunit vaccine platforms are relatively safer than live-attenuated vaccines but have a reduced capacity to promote immune responses. In contrast, viral-vector-based vaccines can trigger strong immune responses and present variable sized antigen proteins; however, there are still safety issues when adopting vectored vaccines.

The current vaccine platforms have used the S protein as an antigen for producing SARS-CoV-2 neutralizing antibodies. The S protein binds to the ACE2 receptor on the alveolar epithelial cells, whose binding motif has been evolutionally conserved [19], and is believed to play an important role in COVID-19 pathogenesis. Moreover, the S protein was shown to represent a good antigen as it can trigger humoral and cellular immune responses. However, the exact molecular and cellular mechanism of COVID-19 remain to be clarified. Recent reports suggest that other viral proteins, such as the E protein and ORF7a accessory protein, may also play an important role [11,12], as they may contribute to the infiltration of the virus into human cells and to inducing serious cytokine storms. Therefore, we investigated the possibility of using engineered S proteins as potential neutralizing antigens. Indeed, our findings suggest that these proteins were able to generate Ag-specific antibodies and invoke Ag-specific cellular immune responses. 

Viral-vector- and mRNA-based COVID-19 vaccines are associated with higher frequency of side effects compared with traditional vaccines, such as the vaccinia virus vaccine and measles vaccine, among others [20]. These side effects appear to be caused by the overdose of lipid nanoparticles (the delivery system used) for introducing the antigen genes into the muscle cells or immunostimulant (adjuvant) substances for enhancing the specific immunity [14]. Therefore, a new type of vaccine platform with fewer side effects than the current ones being used is still warranted.

*Salmonella* strains are known to be a good platform to ensure continuous, long-term antigen protein production owing to its type III secretion system [21,22]. *Salmonella* is an intracellular infectious bacterium and can present antigens during intracellular parasitization by infecting mucosal cells. In particular, the antigen was fused to the SipB signal peptide for effective expression in cells, as shown in the results of research on anticancer drugs that induce cellular immunity [23]. Therefore, although it is a bacterium, it has an infection mechanism similar to that of a virus, and thus, it has the characteristics of inducing mucosal immunity and the expression of antigens (e.g., MHC class I) in cells. This orally administered vaccine has the advantage of inducing more effective cellular and humoral immune responses against the virus using this strain.

*Salmonella*-based vaccines can be administrated via different routes including by oral or intranasal administration [24]. Although orally administrated bacteria-based vaccines can be inactivated by the gut microbiome, they are expected to hold a reduced risk for adjuvant- or virus vector-related adverse side effects. Some reports have suggested that *Salmonella* strains have the characteristic of regulated delayed attenuation when administrated orally [25], and that they can activate long-lasting humoral and cellular immunity [26]. Additionally, the production cost of attenuated *Salmonella*-based vaccines is very low. Currently, the development of anti-coronavirus vaccines using *Salmonella* is insignificant; thus, this report may pave the way for future COVID-19 vaccines.

## 5. Conclusions

This study showed that it is possible to develop safer engineered *Salmonella* strains producing and expressing antigens of various sizes with fewer inflammatory responses, and these could serve as vaccine platforms associated with reduced/negligible toxicity and effective antibody production potential when administered orally. Importantly, this engineered *Salmonella*-based vaccine strategy has the ability to induce antigen-specific T-cell activity and CTL responses, along with antiviral immune responses (such as interferon-γ) without the need for other immunoactive agents. Taken together, *Salmonella* can be considered as a safer oral vaccine development platform for new infectious diseases including COVID-19.

## Supplementary Materials

The following supporting information can be downloaded at: https://www.mdpi.com/article/10.3390/vaccines10010067/s1, Figure S1: Construction of *Salmonella* Typhimurium expressing engineered proteins of SARS-CoV-2 as an antigen. Figure S2: In vivo effects of *S*. Typhimurium expressing Ag.e.spike. Figure S3: Inflammatory cytokine anlysis in mice. Mice were treated with PBS or 1 × 10^7^ bacteria cells. Figure S4: In vivo effects of oral treatment with *S*. Typhimurium expressing Ag-spike.
