# Supplementary material for: Development of an Oral Salmonella-Based Vaccine Platform against SARS-CoV-2
Source: Vaccines (Basel). 2022 Jan 1;10(1):67. doi: 10.3390/vaccines10010067 (PMC8777945; doi:10.3390/vaccines10010067)
Supplement: Supplementary file 1 [file vaccines-10-00067-s001.zip › vaccines-1516968-supplementary.pdf]

Supplement Figure S1

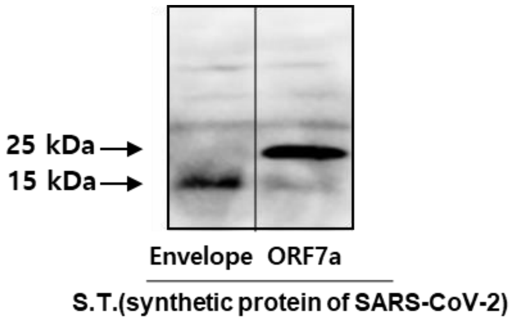

Supplementary Figure S1. Construction of *Salmonella* Typhimurium expressing engineered proteins of SARS-CoV-2as an antigen. Expression of engineered protein (Envelop and ORF7a) of SARS-CoV-2 in genetically engineered *S.* Typhimurium was measured by western blot.

Supplement Figure S2

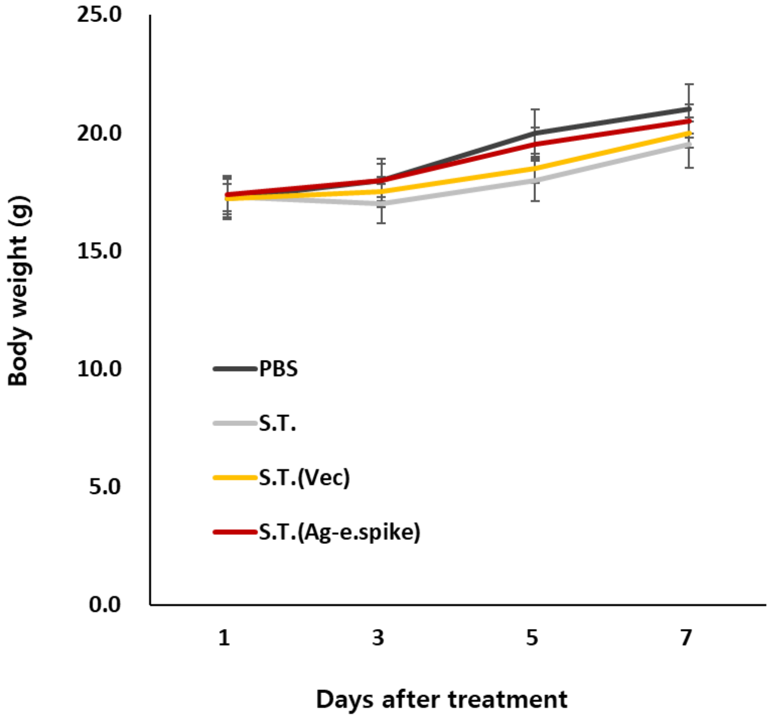

Supplementary Figure S2. *In vivo* effects of *S.* Typhimurium expressing Ag.e.spike. Body weight change was examined in mice after oral administration 1 × 10^7 bacterial cells for the indicated time.

Supplement Figure S3.

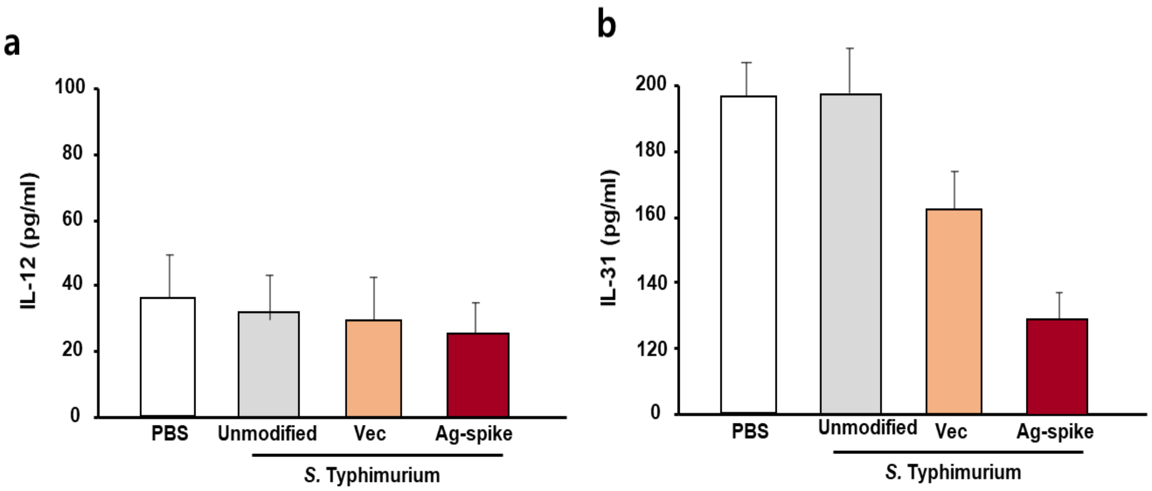

Supplementary Figure S3. Inflammatory cytokine anlysis in mice. Mice were treated with PBS or 1 × 10^7 bacteria cells. Serum was collected 7 days after inoculation and Cytokines (IL-12, IL-31) levels were analyzed with enzyme-linked immunosorbent assay (ELISA).

Supplement Figure S4.

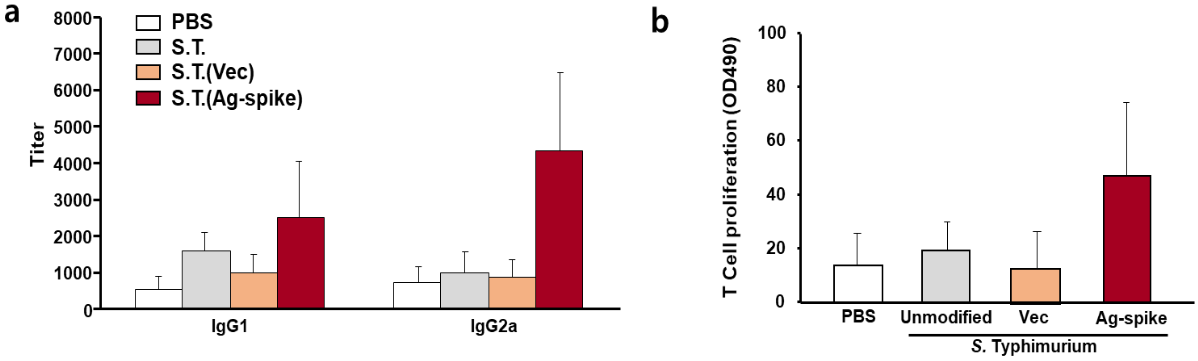

Supplementary Figure S4. *In vivo* effects of oral treatment with *S.* Typhimurium expressing Ag-spike. Balb/c mice were orally inoculated on days 1, 14, and 28 with PBS or with 1 × 10^7 CFU of attenuated unmodified *S.* Typhimurium, *S.* Typhimurium carrying the empty expression vector (vec), or *S. Typhimurium* expressing Ag-spike. (**a**) Sera at 6 week were reacted with *S.* Typhimurium/Ag-spike to determine IgG1 and IgG2a isotype titers. (**b**) T-cell proliferation in mouse spleen cells after orally immunization. CD4 T cells from mouse after oral inoculation with 10^7 CFU *S.* Typhimuriums were activated by spike protein and tests by MTT cell proliferation assay.
